# MFG-E8 Maintains Cellular Homeostasis by Suppressing Endoplasmic Reticulum Stress in Pancreatic Exocrine Acinar Cells

**DOI:** 10.3389/fcell.2021.803876

**Published:** 2022-01-14

**Authors:** Yifan Ren, Wuming Liu, Jia Zhang, Jianbin Bi, Meng Fan, Yi Lv, Zheng Wu, Yuanyuan Zhang, Rongqian Wu

**Affiliations:** ^1^ National Local Joint Engineering Research Center for Precision Surgery and Regenerative Medicine, Shaanxi Provincial Center for Regenerative Medicine and Surgical Engineering, First Affiliated Hospital of Xi’an Jiaotong University, Xi’an, China; ^2^ Department of General Surgery, The Second Affiliated Hospital of Xi’an Jiaotong University, Xi’an, China; ^3^ Department of Hepatobiliary Surgery, First Affiliated Hospital of Xi’an Jiaotong University, Xi’an, China; ^4^ Department of Oncology, The Second Affiliated Hospital of Xi’an Jiaotong University, Xi’an, China; ^5^ Department of Pediatrics, First Affiliated Hospital of Xi’an Jiaotong University, Xi’an, China

**Keywords:** pancreatic exocrine acinar cells, endoplasmic reticulum stress, acute pancreatitis, MFG-E8, αvβ3/5 integrins, FAK-STAT3 pathway

## Abstract

Excessive endoplasmic reticulum (ER) stress contributes significantly to the pathogenesis of exocrine acinar damage in acute pancreatitis. Our previous study found that milk fat globule EGF factor 8 (MFG-E8), a lipophilic glycoprotein, alleviates acinar cell damage during AP via binding to αvβ3/5 integrins. Ligand-dependent integrin-FAK activation of STAT3 was reported to be of great importance for maintaining cellular homeostasis. However, MFG-E8’s role in ER stress in pancreatic exocrine acinar cells has not been evaluated. To study this, thapsigargin, brefeldin A, tunicamycin and cerulein + LPS were used to induce ER stress in rat pancreatic acinar cells *in vitro*. L-arginine- and cerulein + LPS-induced acute pancreatitis in mice were used to study ER stress *in vivo*. The results showed that MFG-E8 dose-dependently inhibited ER stress under both *in vitro* and *in vivo* conditions. MFG-E8 knockout mice suffered more severe ER stress and greater inflammatory response after L-arginine administration. Mechanistically, MFG-E8 increased phosphorylation of FAK and STAT3 in cerulein + LPS-treated pancreatic acinar cells. The presence of specific inhibitors of αvβ3/5 integrin, FAK or STAT3 abolished MFG-E8’s effect on cerulein + LPS-induced ER stress in pancreatic acinar cells. In conclusion, MFG-E8 maintains cellular homeostasis by alleviating ER stress in pancreatic exocrine acinar cells.

## Graphical Abstract

**Figure:**
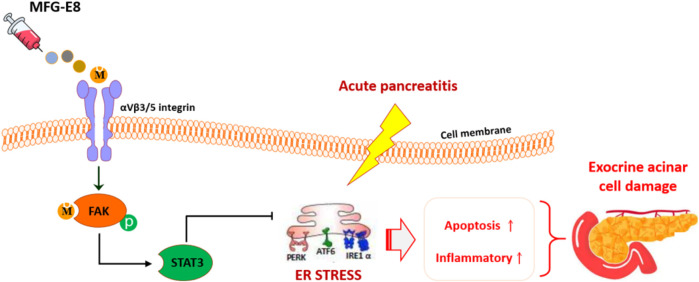
MFG-E8 alleviate ER stress in pancreatic exocrine acinar cells via αVβ3/5 integrin-FAK-STAT3 pathway.

## Introduction

Endoplasmic reticulum (ER) stress is involved in the damage of pancreatic exocrine acinar cells in acute pancreatitis (Sun et al., 2019; Tan et al., 2020). Inositol-required enzyme 1 (IRE1)-XBP1, PKR-like endoplasmic reticulum kinase (PERK), eukaryotic initiation factor 2 alpha (eIF2α), activating transcription factor 6 (ATF6), and binding immunoglobulin protein (BIP, also known as glucose-regulated protein 78-kDa, GRP78) are the main signaling pathways that mediate the development of ER stress (Jiang et al., 2010; He, 2021; You et al., 2021). ER stress induced by different causes activates these pathways, respectively or simultaneously, and leads to the unfolded protein response (UPR). UPR participates in the adaptation to various stimuli and restores cellular homeostasis. However, under excessive ER stress conditions, UPR could not further alleviate cellular damage, but instead accelerate cell death by inducing the activation of apoptosis-related molecules transcription factor C/EBP homologous protein (CHOP) and caspase-9 (Gorman et al., 2012). CHOP and caspase-9 reduce the level of Bcl-2 and activate caspase-3 (Li et al., 2014; Datta et al., 2018), which ultimately leads to apoptosis of the cell. When ER stress occurs, IRE1α recruits TRAF2 into ER and initiates the inflammatory response via activating the NF-κB pathway (Keestra-Gounder et al., 2016). The storm of the inflammatory response not only sweeps through the affected organs, but also induces systemic damage, resulting in multiple organ injury.

Milk fat globule EGF factor 8 (MFG-E8), a secreted lipophilic glycoprotein, contains an RGD motif and interacts with integrins (Yang et al., 2011; An et al., 2017). It participates in a wide range of cellular communications, such as mediating and maintaining the binding between the sperm and epididymal epithelial cells, repairing intestinal epithelial cells, promoting angiogenesis, and enhancing the phagocytosis and clearance of apoptotic cells by macrophages (Hanayama et al., 2002; Miksa et al., 2008; Kranich et al., 2010; Aziz et al., 2015). Our previous studies have demonstrated that MFG-E8 alleviates pancreatic tissue damage during acute pancreatitis via binding to αvβ3/5 integrins (Ren et al., 2021a). Ligand-dependent integrin-FAK activation of STAT3 was reported to be of great importance for maintaining cellular homeostasis (Banerjee et al., 2017). However, whether MFG-E8 also has any effects on ER stress in pancreatic exocrine acinar cells remains largely unknown. Therefore, in this study, we aimed to clarify the specific role and molecular mechanism of MFG-E8 in ER stress of pancreatic exocrine acinar cells during acute pancreatitis.

## Results

### MFG-E8 Alleviates ER Stress in Pancreatic Exocrine Acinar Cells

**FIGURE 1:**
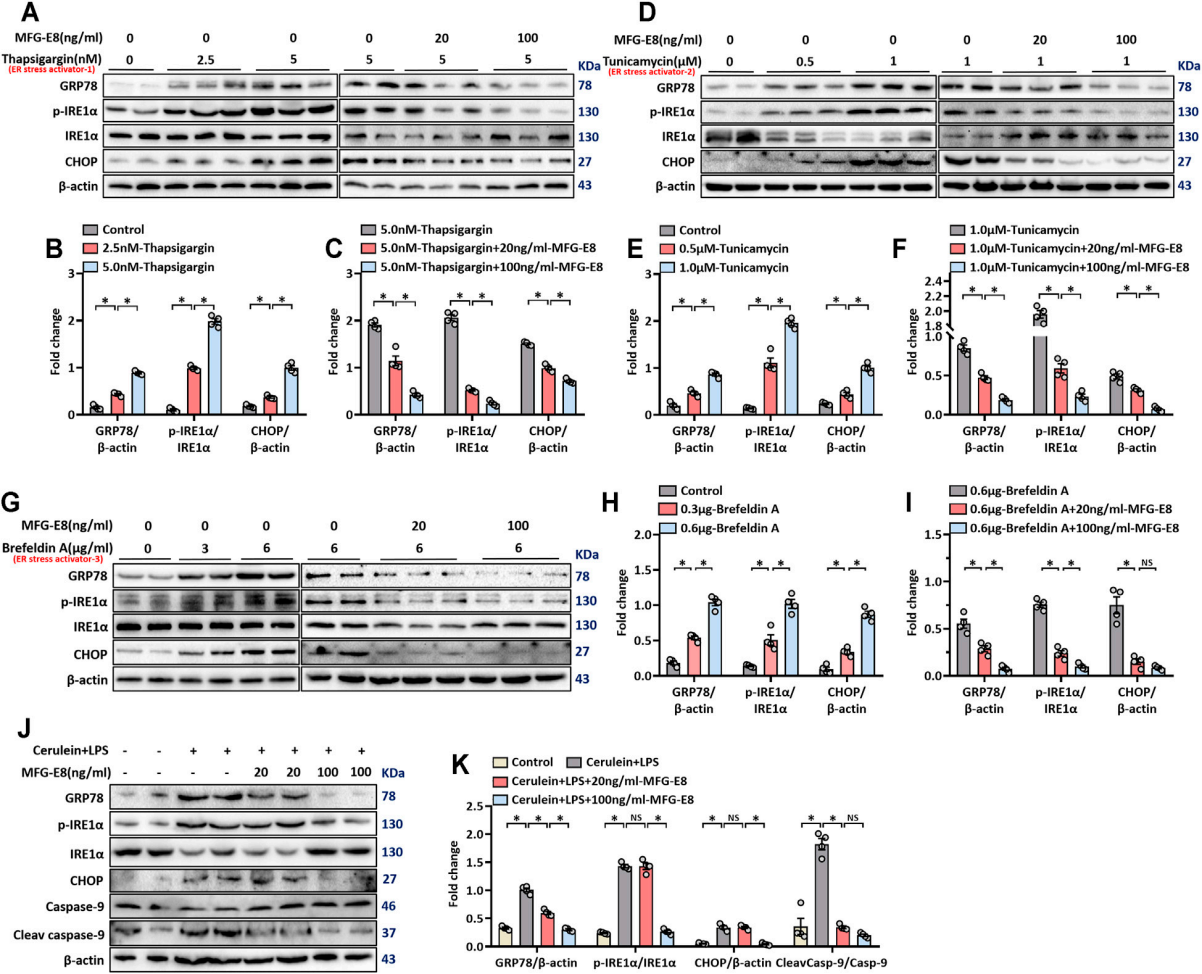
rhMFG-E8 alleviates ER stress in pancreatic exocrine acinar cells. Model 1: Pancreatic AR42J cells (5 × 10^5^/well) were treated with 2.5 or 5 nM thapsigargin with or without 20 or 100 ng/ml MFG-E8 for 24 h. Model 2: Pancreatic AR42J cells (5 × 10^5^/well) were treated with 0.5 or 1 μM tunicamycin with or without 20 or 100 ng/ml MFG-E8 for 24 h. Model 3: Pancreatic AR42J cells (5 × 10^5^/well) were treated with 3 or 6 μg/ml brefeldin A with or without 20 or 100 ng/ml MFG-E8 for 24 h. Model 4: Pancreatic AR42J cells (5 × 10^5^/well) were treated with 100 nmol/L cerulein and 10 ng/ml LPS with or without 20 or 100 ng/ml MFG-E8 for 24 h. **(A–I)** Western blot analysis of the expression of GRP78, phospho-IRE1α, IRE1α and CHOP in the AR42J cells; **(J,K)** Western blot analysis of the expression of GRP78, phospho-IRE1α, IRE1α, CHOP, caspase-9, and cleaved caspase-9 in the AR42J cells. *n* = 4–6/group, error bars indicate the SEM; ∗*p* < 0.05 versus Sham group; #*p* < 0.05 versus Vehicle group. MFG-E8, milk fat globule EGF factor 8; GRP78, glucose-regulated protein 78; CHOP, C/EBP homologous protein; LPS, Lipopolysaccharide.

We used three commonly used ER stress activators thapsigargin, brefeldin A and tunicamycin to induce ER stress in AR42J cells, a rat pancreatic exocrine acinar cell line (Johnson et al., 2009; Misiewicz et al., 2013; Abhari et al., 2019). As shown in Figures 1A–C, 2.5 nM thapsigargin slightly increased the expression levels of GRP78, phospho-IRE1α and CHOP in AR42J cells, 5 nM thapsigargin significantly increased the expression levels of these three ER stress-related proteins in AR42J cells (*p* < 0.05). Similarly, the expression levels of GRP78, phospho-IRE1α and CHOP in AR42J cells also showing a gradual increase in the increase of tunicamycin (*p* < 0.05, Figures 1D–F) and brefeldin A (*p* < 0.05, Figures 1G–I) doses. Next, we added 20 ng/ml and 100 ng/ml of recombinant MFG-E8 to AR42J cells treated with 5 nM thapsigargin, 1 μM tunicamycin, and 6 μg/ml brefeldin A, respectively. It was observed that exogenous MFG-E8 had a significant inhibitory effect on ER stress induced by three ER stress activators, and the inhibitory effect was dose-dependent (*p* < 0.05, Figures 1A–I). Furthermore, the expression level of MFG-E8 in AR42J cells decreased with the increase of the dose of the three ER stress activators (*p* < 0.05, Supplementary Figure S1).

Cerulein + lipopolysaccharide (LPS) are often used to induced acute pancreatitis in rodents and in cultured pancreatic acinar cells (Fu et al., 2020; Xiao et al., 2020). Our previous study found that treatment of AR42J cells with cerulein + LPS induced an obvious stress response in the ER (Ren et al., 2019a). In this study, cerulein + LPS was also used to induce ER stress in AR42J cells and the effect of exogenous MFG-E8 on ER stress was tested. As shown in Figures 1J,K, cerulein + LPS significantly upregulated the expression of GRP78, phospho-IRE1α, CHOP and cleaved-caspase-9, suggesting activation of the ER stress. 100 ng/ml MFG-E8 significantly, while 20 ng/ml MFG-8 partially, inhibited cerulein + LPS induced upregulation of these proteins (Figures 1J,K).

### MFG-E8 Alleviates Pancreatic ER Stress *In Vivo*

**FIGURE 2:**
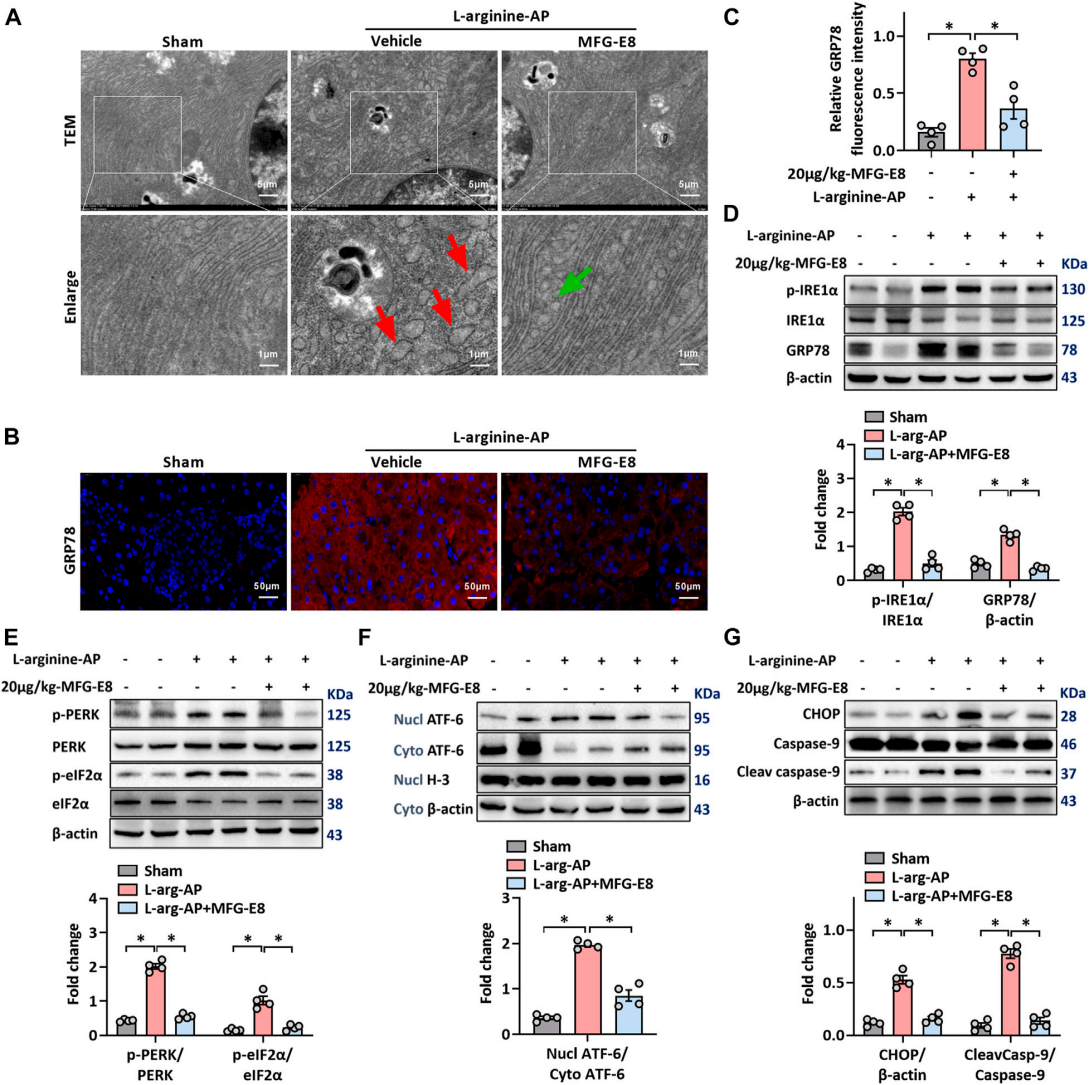
Exogenous MFG-E8 alleviates pancreatic ER stress *in vivo*. In mice, arginine-AP stress was induced by 2 h intraperitoneal injections of 4.0 g/kg L-arginine. At 2 h after the last injection of L-arginine, normal saline (vehicle) or 20 μg/kg MFG-E8 were administered through intraperitoneal injection. The animals were sacrificed at 69 h after MFG-E8 treatment (i.e., 72 h after the first injection of L-arginine). Blood and tissue samples were collected. **(A)** Ultrastructural alterations in the pancreas (Transmission electron microscopy); **(B,C)** Representative photos of GRP78 staining and quantitative of GRP78 staining; **(D)** Western blot analysis of the expression of GRP78, phospho-IRE1α and IRE1α in the pancreas; **(E)** Western blot analysis of the expression of phospho-PERK, PERK, phospho-eIF2α and eIF2α in the pancreas; **(F)** Western blot analysis of the expression of nucl-ATF-6, cyto-ATF-6, nucl-H3 and cyto-β-actin in the pancreas; **(G)** Western blot analysis of the expression of CHOP, caspase-9 and cleaved caspase-9 in the pancreas. *n* = 4–6/group, error bars indicate the SEM; ∗ *p* < .05 *versus* Sham group; #*p* < .05 *versus* Vehicle group. MFG-E8, milk fat globule EGF factor 8; AP, acute pancreatitis; GRP78, glucose-regulated protein 78; eIF2α, eukaryotic initiation factor 2α; ATF-6, Activating Transcription Factor 6; CHOP, C/EBP homologous protein; Nucl, nucleus; Cyto, cytoplasm; H-3, histone-3; PERK, PKR-like endoplasmic reticulum kinase.

L-arginine-induced acute pancreatitis in mice causes extensive ER stress in exocrine acinar cells (Biczo et al., 2018; Ren et al., 2019a; Ren et al., 2021a), and we used this animal model to test whether exogenous MFG-E8 also alleviates ER stress *in vivo*. TEM showed swelling, fragmentation, vacuolization and disintegration of the ER in the pancreatic tissue after L-arginine treatment (Figure 2A). Intraperitoneal injection of exogenous MFG-E8 reduced ER damage in pancreatic acinar cells of L-arginine-treated mice. Immunofluorescence staining and western blot analysis showed that intraperitoneal injection of 20 μg/kg body weight MFG-E8 significantly reduced the expression of GRP78 in the pancreatic tissue of L-arginine-treated mice (Figures 2B–D, *p* < 0.05). The expression of phospho-IRE1α was also decreased after exogenous MFG-E8 injection (*p* < 0.05, Figure 2D).

The PERK-eIF2α and ATF-6 pathways are the other two of the three classical pathways of ER stress, phosphorylation of PERK and eIF2α or cytoplasmic ATF-6 translocation into the nucleus indicate activation of these two ER stress pathways (Kubisch et al., 2006). The intraperitoneal injection of 20 μg/ml-MFG-E8 also inhibited the activation of these two pathways in mouse pancreatic tissue, suggesting that exogenous MFG-E8 could effectively inhibit L-arginine-induced ER stress in pancreatic tissue through multiple pathways (*p* < 0.05, Figures 2E,F). Similarly, exogenous MFG-E8 also reduced the expression levels of ER stress-related apoptotic proteins CHOP and cleaved caspase-9 (*p* < 0.05, Figure 2G), and the anti-apoptotic effect of MFG-E8 may also be realized through this pathway. Similar to the *in vitro* experiments, the reduction of ER stress in pancreatic tissues of AP mice by exogenous MFG-E8 was also dose-dependent. Compared with 10 μg/kg, 20 μg/kg MFG-E8 almost completely inhibited the expression of ER stress-related proteins in pancreatic tissues of AP mice (Supplementary Figure S2).

Intraperitoneal injection of 20 μg/kg MFG-E8 also alleviated ER stress of pancreatic cells in cerulein + LPS-treated mice (*p* < 0.05, Supplementary Figures S3A–D).

### MFG-E8 Alleviates the Inflammatory Response in Experimental-AP Through NF-κB Signaling Pathway

**FIGURE 3:**
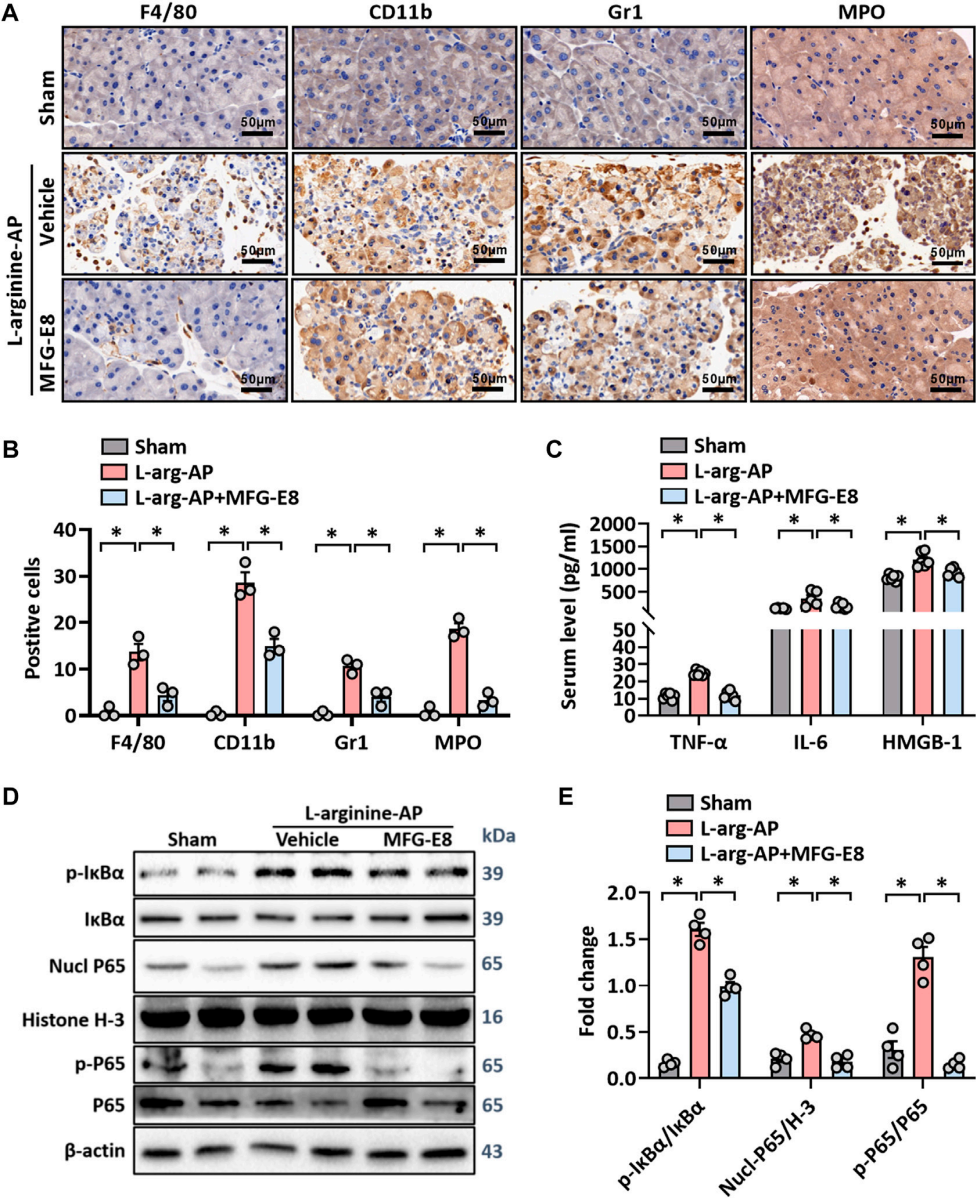
MFG-E8 alleviates the inflammatory response in experimental-AP through NF-κB signaling pathway. In mice, arginine-AP stress was induced by 2 h intraperitoneal injections of 4.0 g/kg L-arginine. At 2 h after the last injection of L-arginine, normal saline (vehicle) or 20 μg/kg MFG-E8 were administered through intraperitoneal injection. The animals were sacrificed at 69 h after MFG-E8 treatment (i.e., 72 h after the first injection of L-arginine). Blood and tissue samples were collected. **(A)** Representative photos of F4/80, CD11b, Gr1 and MPO staining; **(B)** Quantitative of F4/80, CD11b, Gr1 and MPO staining; **(C)** Serum TNF-α, IL-6 and HMGB-1 levels; **(D,E)** Western blot analysis of the expression of phospho-IκBα, IκBα, nucl-P65, cyto-P65, nucl-H3 and cyto-β-actin in the pancreas. *n* = 4–6/group, error bars indicate the SEM; ∗ *p* < .05 *versus* Sham group; #*p* < .05 *versus* Vehicle group. MFG-E8, milk fat globule EGF factor 8; AP, acute pancreatitis; MPO, myeloperoxidase; HMGB-1, High mobility group box 1; Nucl, nucleus; Cyto, cytoplasm; H-3, histone-3; IκBα, inhibitor of NF-κB-α; NF-κB p65, Nuclear Factor Kappa-B p65.

Unresolvable ER stress leads to inflammatory responses (Eugene et al., 2020) and inflammation is another pathological feature of L-arginine-induced AP (El Morsy and Ahmed, 2020; Abdelzaher et al., 2021). As shown in Figures 3A,B, immunohistochemical staining of F4/80 and CD11b showed that there was a large amount of macrophage infiltration in the pancreatic tissue of L-arginine treated mice, and intraperitoneal injection of 20 μg/kg-MFG-E8 effectively reduced the degree of macrophage infiltration (*p* < 0.05). Gr1 and MPO-labeled neutrophils showed the same trend as macrophages, suggesting that exogenous MFG-E8 effectively reduced the number of inflammatory cells in the pancreatic tissue (*p* < 0.05, Figures 3A,B). Serum levels of TNF-α, IL-6 and HMGB1 also indicated that exogenous MFG-E8 had a significant anti-inflammatory effect in experimental-AP (*p* < 0.05, Figure 3C).

Activation of IKK leads to the phosphorylation and isolation of inhibitor of NF-κB-α (IκBα), which is bound to Nuclear Factor Kappa-B p65 (NF-κB p65). The dissociated NF-κB p65 is transferred from the cytoplasm to the nucleus and binds with the corresponding inflammation-related genes to initiate the transcription of inflammatory cytokines and induce inflammation (Sun, 2017; Zhang et al., 2017). As shown in Figures 3D,E, phospho-IκBα was significantly increased in L-arginine-treated mice (*p* < 0.05). Accordingly, nucleus NF-κB p65 and phospho-NF-κB p65 also increased, suggesting that NF-κB p65 dissociated and translocated into the nucleus from the cytoplasm in L-arginine-treated mice (*p* < 0.05, Figures 3D,E). The intraperitoneal injection of exogenous MFG-E8 significantly reduced the phospho-IκBα, nucleus NF-κB p65 and phospho-NF-κB p65 expression levels in L-arginine-treated mice.

### MFG-E8 Deficiency Aggravated Pancreatic ER Stress and Inflammation in L-Arginine-Treated Mice

**FIGURE 4:**
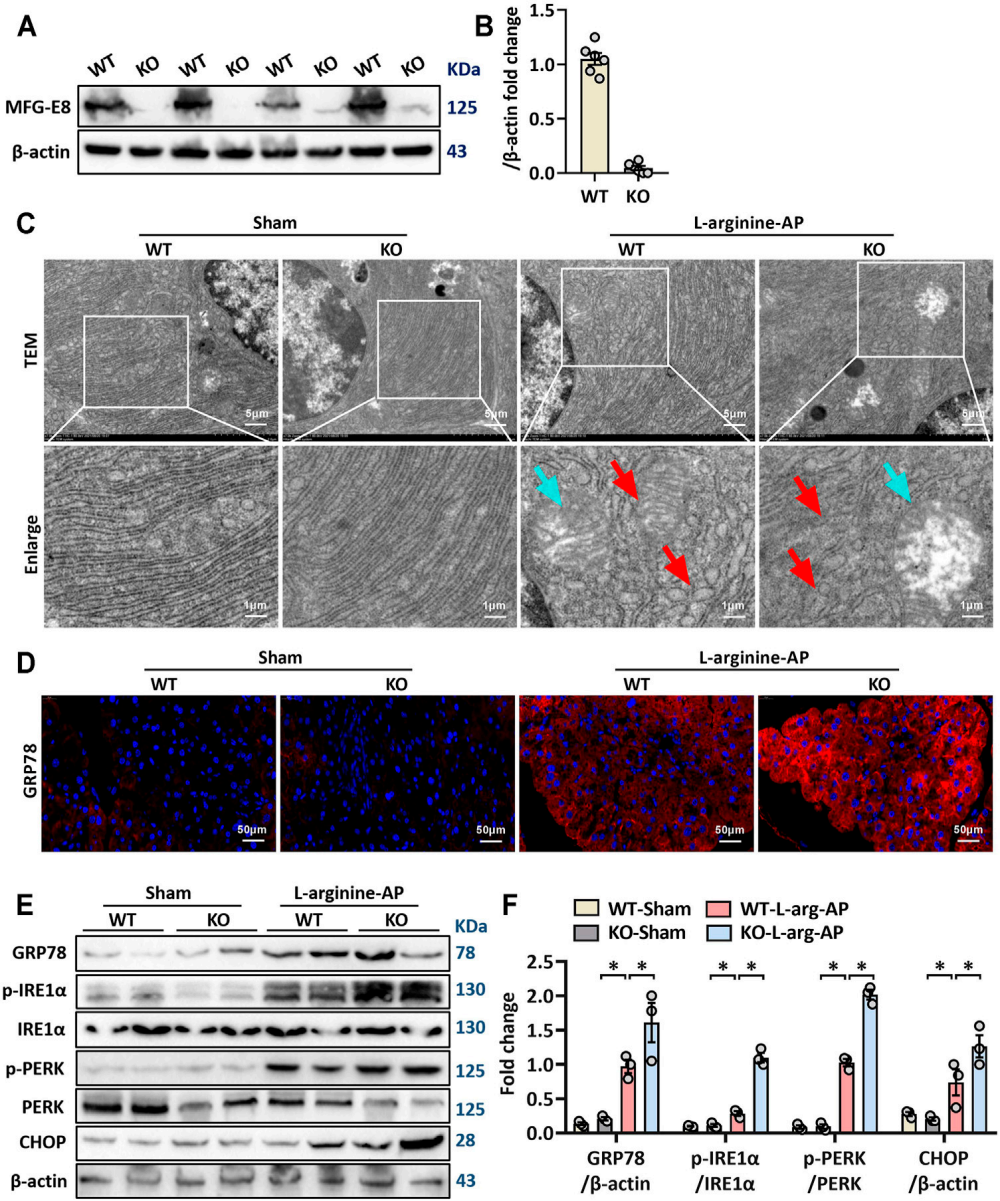
MFG-E8 deficiency aggravated pancreatic ER stress in L-arginine-treated mice. In mice, arginine-AP stress was induced by 2 hourly intraperitoneal injections of 4.0 g/kg L-arginine. At 2 h after the last injection of L-arginine, normal saline (vehicle) or 20 μg/kg MFG-E8 were administered through intraperitoneal injection. The animals were sacrificed at 69 h after MFG-E8 treatment (i.e., 72 h after the first injection of L-arginine). Blood and tissue samples were collected. **(A,B)** Western blot analysis of the expression of MFG-E8 in the pancreas; **(C)** Ultrastructural alterations in the pancreas (Transmission electron microscopy); **(D)** Representative photos of GRP78 staining; **(E,F)** Western blot analysis of the expression of GRP78, phospho-PERK, PERK, phospho-IRE1α, IRE1α and CHOP in the pancreas. *n* = 4–6/group, error bars indicate the SEM; ∗*p* < 0.05 versus Sham group; #*p* < 0.05 versus Vehicle group. WT, wild type; KO, knockout; MFG-E8, milk fat globule EGF factor 8; AP, acute pancreatitis; GRP78, glucose-regulated protein 78; CHOP, C/EBP homologous protein; PERK, PKR-like endoplasmic reticulum kinase.

**FIGURE 5:**
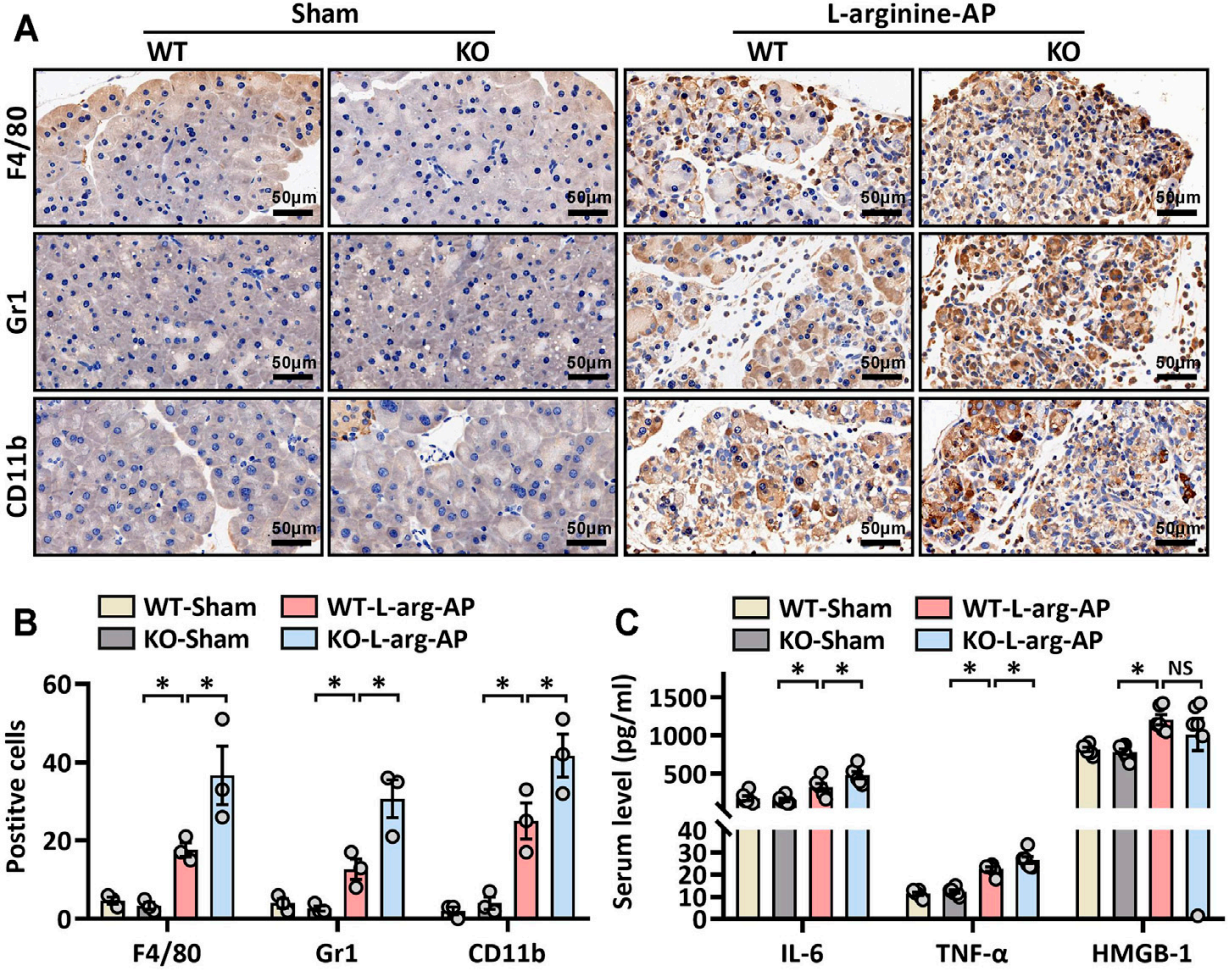
MFG-E8 deficiency aggravated pancreatic inflammatory response in L-arginine-treated mice. In mice, arginine-AP stress was induced by 2 hourly intraperitoneal injections of 4.0 g/kg L-arginine. At 2 h after the last injection of L-arginine, normal saline (vehicle) or 20 μg/kg MFG-E8 were administered through intraperitoneal injection. The animals were sacrificed at 69 h after MFG-E8 treatment (i.e., 72 h after the first injection of L-arginine). Blood and tissue samples were collected. **(A)** Representative photos of F4/80, CD11b, and Gr1 staining; **(B)** Quantitative of F4/80, CD11b, and Gr1 staining; **(C)** Serum TNF-α, IL-6 and HMGB-1 levels. *n* = 4–6/group, error bars indicate the SEM; ∗*p* < 0.05 versus Sham group; #*p* < 0.05 versus Vehicle group. WT, wild type; KO, knockout; MFG-E8, milk fat globule EGF factor 8; AP, acute pancreatitis; HMGB-1, High mobility group box 1.

We then further investigated the role of MFG-E8 in ER stress in pancreatic tissue using MFG-E8 knockout (*mfge8*-KO) mice. As shown in Figures 4A,B, compared with the wild-type mice of the same litter, the expression level of MFG-E8 in the pancreas of *mfge8*-KO mice almost completely disappeared (*p* < 0.05). We induced ER stress in mouse pancreatic tissues with L-arginine, and found that *MFG-E8 deficiency* aggravated ER injury (red arrow) and exacerbated mitochondrial morphological abnormalities (blue arrow) (Figure 4C). *MFG-E8 deficiency* also upregulated GRP78 expression (*p* < 0.05, Figures 4D–F, immunofluorescence staining and western blotting). Similarly, phospho-IRE1α, phospho-PERK, and CHOP were also higher in *mfge8*-KO mice, suggesting that the presence of MFG-E8 in pancreatic tissue plays a role in limiting the severity of ER stress (*p* < 0.05, Figures 4E,F). The infiltration level of inflammatory cells in pancreatic tissue (neutrophils labeled by Gr1; macrophages labeled by F4/80 and CD11b) and the number of inflammatory cells released into serum were also significantly higher in *mfge8*-KO mice (*p* < 0.05, Figures 5A–C). The above evidence shows that the presence of MFG-E8 has a considerable effect on alleviating L-arginine-induced ER stress and inflammation of pancreatic tissue.

### MFG-E8 Alleviates ER Stress Through the Integrin αVβ3/5-FAK-STAT3 Pathway

**FIGURE 6:**
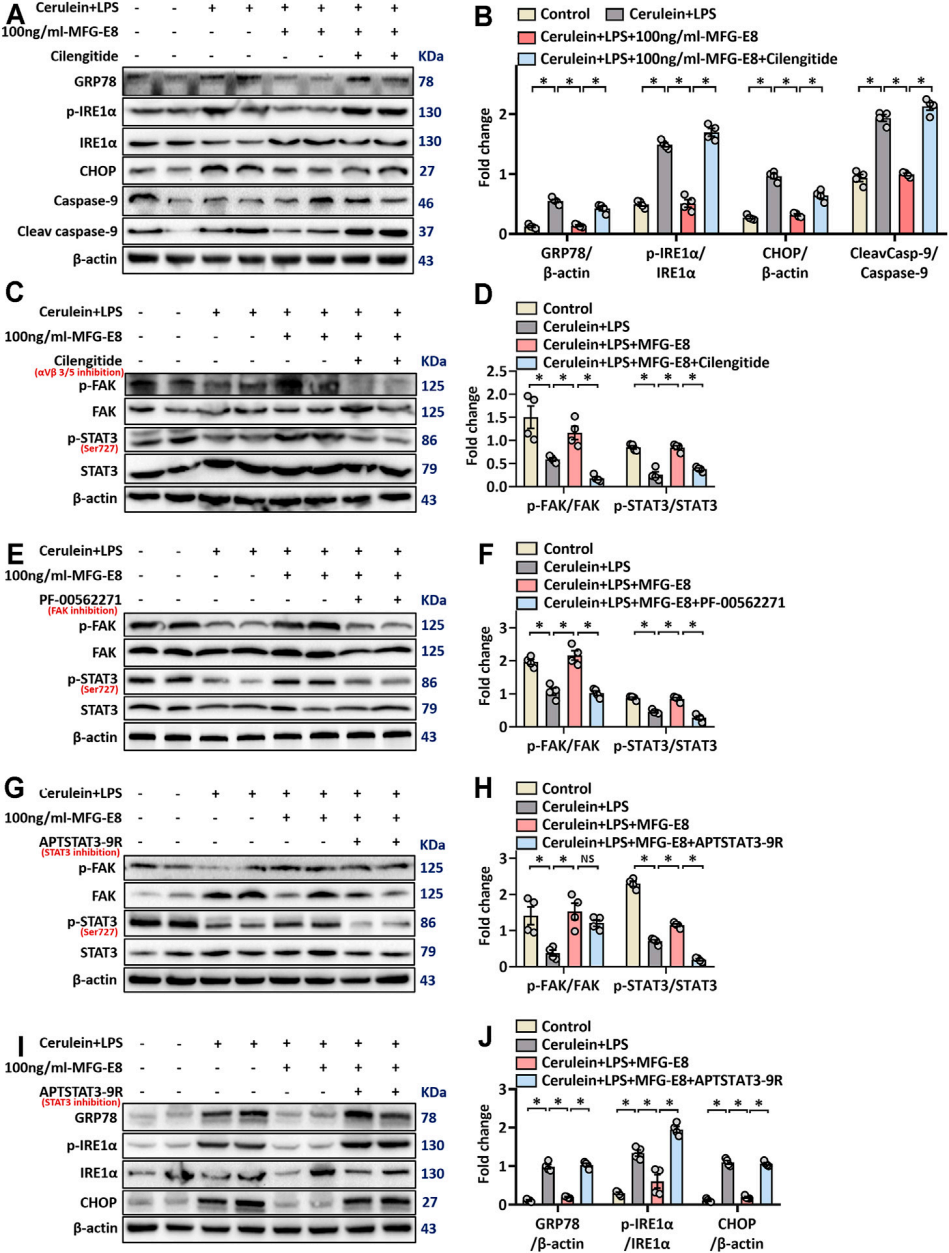
rhMFG-E8 alleviates ER stress in pancreatic exocrine acinar cells through integrin αVβ3/5-FAK-STAT3. Pancreatic AR42J cells (5 × 10^5^/well) were treated with 100 nmol/L cerulein and 10 ng/ml LPS with or without 100 ng/ml MFG-E8 for 24 h. To determine whether the protective effect of MFG-E8 in ER stress is mediated through integrin αVβ3/5-FAK-STAT3, 5 ng/ml cilengitide, a specific integrin αVβ3/5 antagonist, 1 μmol/L-PF-00562271, a specific FAK antagonist, 30 μmol/L-APTSTAT3-9R, a specific STAT3 antagonist, was administered with 100 ng/ml MFG-E8, respectively. **(A,B)** Western blot analysis of the expression of GRP78, phospho-IRE1α, IRE1α, CHOP, caspase-9 and cleaved caspase-9 in the AR42J cells; **(C–H)** Western blot analysis of the expression of phospho-FAK, FAK, phospho-STAT3 and STAT3 in the AR42J cells; **(I,J)** Western blot analysis of the expression of GRP78, phospho-IRE1α, IRE1α, CHOP, caspase-9 and cleaved caspase-9 in the AR42J cells. *n* = 6/group, error bars indicate the SEM; ∗ *p* < 0.05 versus Sham group; #*p* < 0.05 versus Vehicle group. MFG-E8, milk fat globule EGF factor 8; GRP78, glucose-regulated protein 78; CHOP, C/EBP homologous protein; LPS, Lipopolysaccharide.

Our previous study found that the biological effects of exogenous MFG-E8 require binding to the integrin αVβ3/5 receptor (Ren et al., 2021a). Whether MFG-E8 alleviates ER stress also acts on integrin αVβ3/5 remains unknown. Therefore, we utilized cilengitide, a highly effective and specific integrin αVβ3/5 inhibitor (Wang et al., 2020), to test whether the effect of MFG-E8 on ER stress is also mediated by this receptor. As shown in Figures 6A,B, the addition of cilengitide almost completely antagonized the effect of 100 ng/ml MFG-E8 on AR42J cell ER stress (*p* < 0.05).

Ligand-dependent integrin-FAK activation of STAT3 was reported to be of great importance for maintaining cellular homeostasis (Wu et al., 2020). To explore whether the effect of MFG-E8 on ER stress of pancreatic exocrine acinar cells is also mediated through this pathway, we detected the levels of P-FAK and P-STAT3 in AR42J cells. As shown in Figures 6C,D, 100 ng/ml-MFG-E8 effectively restored the decrease of levels of P-FAK and P-STAT3 in AR42J cells induced by cerulein + LPS (*p* < 0.05). The addition of cilengitide antagonized the effect of MFG-E8 on P-FAK and P-STAT3, suggesting that exogenous MFG-E8 affected phosphorylation of FAK and STAT3 via binding to integrin αVβ3/5 (*p* < 0.05, Figures 6C,D).

To further explore the relationship between FAK and STAT3 signal transduction, we applied specific FAK inhibitors and specific STAT3 inhibitors, respectively. As shown in Figures 6E–H, PF-00562271, a specific FAK inhibitor, almost completely antagonized the effects of MFG-E8 against P-FAK and P-STAT3 (*p* < 0.05). However, APTSTAT3-9R, a specific STAT3 inhibitor, only eliminated the effect of MFG-E8 on P-STAT3, but did not prevent the effect of MFG-E8 on P-FAK (*p* > 0.05). We then examined GRP78, phosphorylated IRE1α, CHOP, and cleaved caspase-9 levels and found that APTSTAT3-9R also inhibited the effect of MFG-E8 on cerulein + LPS-induced ER stress in pancreatic acinar cells (*p* < 0.05, Figures 6I,J). These results suggest that exogenous MFG-E8 suppresses ER stress via activating the integrin αVβ3/5-FAK-STAT3 signaling pathway in pancreatic acinar cells.

**FIGURE 7:**
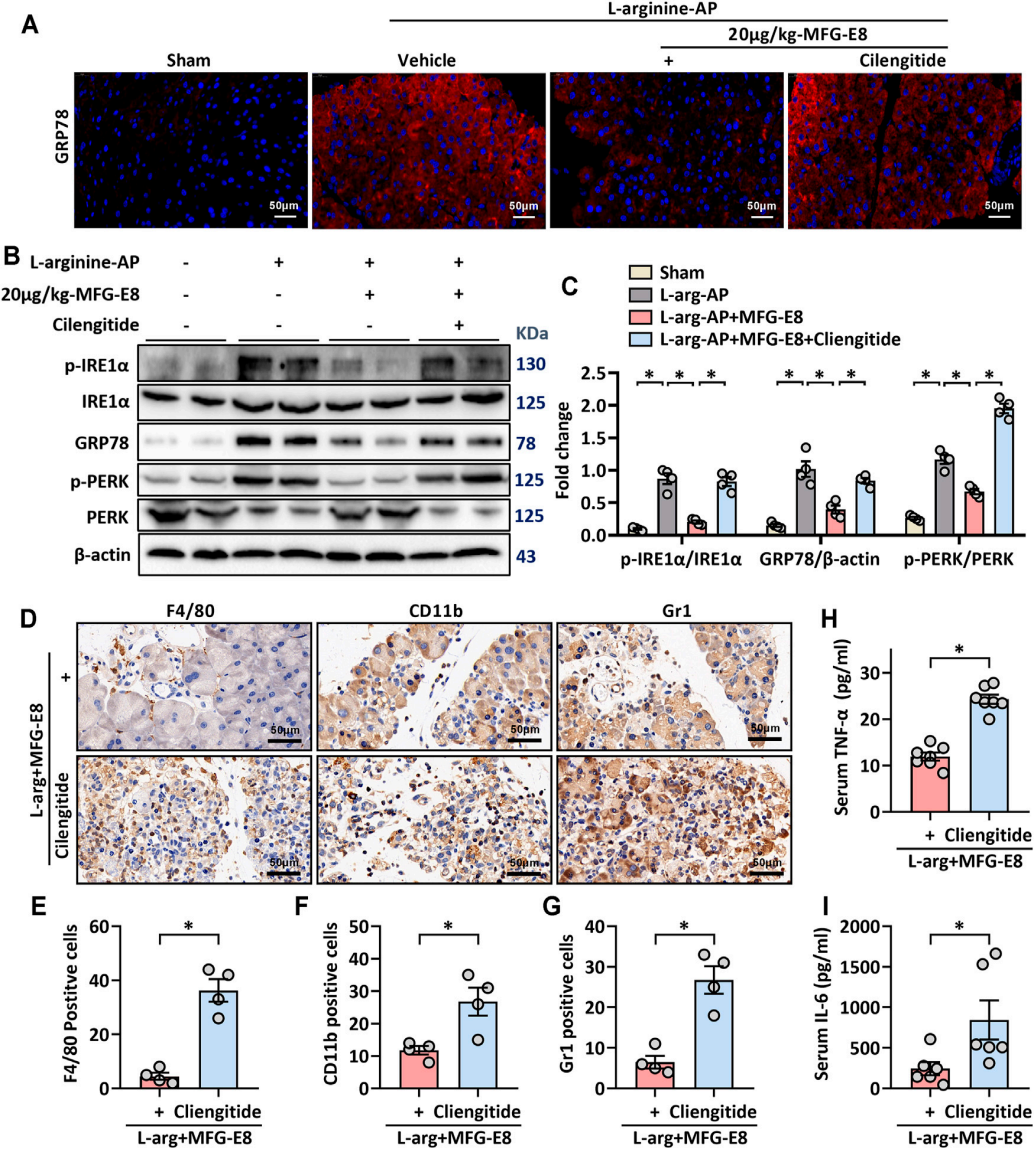
MFG-E8 alleviates ER stress and pancreatic inflammatory response by binding to integrin αVβ3/5 in experimental AP. In mice, arginine-AP stress was induced by 2 hourly intraperitoneal injections of 4.0 g/kg L-arginine. At 2 h after the last injection of L-arginine, normal saline (vehicle) or 20 μg/kg MFG-E8 were administered through intraperitoneal injection. The animals were sacrificed at 69 h after MFG-E8 treatment (i.e., 72 h after the first injection of L-arginine). Blood and tissue samples were collected. **(A)** Representative photos of GRP78 staining; **(B,C)** Western blot analysis of the expression of GRP78, phospho-PERK, PERK, phospho-IRE1α, IRE1α and CHOP in the pancreas; **(D)** Representative photos of F4/80, CD11b and Gr1 staining; **(E–G)** Quantitative of F4/80, CD11b, and Gr1 staining; **(H)** Serum IL-6 levels; **(I)** Serum IL-6 levels. *n* = 4–6/group, error bars indicate the SEM; ∗ *p* < 0.05 versus Sham group; #*p* < 0.05 versus Vehicle group. MFG-E8, milk fat globule EGF factor 8; AP, acute pancreatitis; GRP78, glucose-regulated protein 78; CHOP, C/EBP homologous protein; PERK, PKR-like endoplasmic reticulum kinase.

Consistent with the *in vitro* results, our *in vivo* study also showed that cilengitide antagonized the effect of MFG-E8 on GRP78 (*p* < 0.05, Figures 7A–C). Similarly, the effect of MFG-E8 on the reduction of phospho-IRE1α, phospho-PERK, and CHOP was almost eliminated by cilengitide (*p* < 0.05, Figures 7B,C).

The effect of MFG-E8 on inflammatory cell infiltration in pancreatic tissue was also decreased with cilengitide intervention. Immunohistochemical staining showed that the inhibition of MFG-E8 on neutrophils (labeled by Gr1) and macrophages (labeled by F4/80 and CD11b) infiltration in the pancreatic tissue was also blocked by cilengitide (*p* < 0.05, Figures 7D–G). And the levels of inflammatory mediators (TNF-α and IL-6) in serum also changed accordingly (Figures 7H,I).

## Discussion

In this study, we found that MFG-E8 suppresses ER stress of pancreatic exocrine acinar cells under both *in vivo* and *in vitro* conditions. MFG-E8 deficiency, on the other hand, aggravates ER stress in experimental acute pancreatitis. The effect of MFG-E8 on ER stress seems to be achieved through activating the integrin αVβ3/5-FAK-STAT3 signaling pathway.

ER stress plays a critical role in the development of acute pancreatitis. Several studies have shown that inhibition of ER stress is beneficial in experimental acute pancreatitis (He et al., 2021; Wu et al., 2021). Thapsigargin, brefeldin A and tunicamycin are commonly used ER stress inducers (Misiewicz et al., 2013; Abhari et al., 2019). In our *in vitro* experiment, we also verified the dose-dependence of rat pancreatic acinar cells on the above three ER stress inducers. Our previous studies have confirmed that administration of cerulein + LPS or L-arginine induces ER stress in pancreatic cells in mice (Ren et al., 2019a; Ren et al., 2021a). In this study, we applied four *in vitro* models and two *in vivo* models to verify the effect of exogenous MFG-E8 on ER stress in pancreatic exocrine acinar cells. It is expected to avoid selection bias as much as possible. These different induction modes cause ER stress through different mechanisms. In this experiment, we found that exogenous MFG-E8 not only inhibited the ER stress of AR42J cells under various *in vitro* conditions in a dose-dependent manner, but also reduced ER stress in L-arginine or cerulein + LPS treated mice.

MFG-E8 is a secreted protein that has been shown to promote apoptotic cell clearance and acts as an anti-inflammatory in a variety of organs (Hanayama et al., 2004; Gao et al., 2021). MFG-E8 deficiency causes the aggravation of systemic sclerosis and increases the degree of skin fibrosis in mice (Fujiwara et al., 2019). Our previous study showed that MFG-E8 activates the FAK-STAT3 signaling pathway and alleviates the extent of mitochondrial damage during acute pancreatitis (Ren et al., 2021a). Mitochondrial dysfunction and ER stress are closely related to each other. Banerjee et al. (2017) reported that inhibition of the FAK-STAT3 signaling pathway contributes to ER stress-induced mitochondrial dysfunction and death in endothelial cells. Meanwhile, Song et al. (2020) found that the increased p-STAT3 expression during chronic stress may promote splenocyte survival. The results from this study suggest that the inhibitory effect of MFG-E8 on ER stress of pancreatic cells during acute pancreatitis is also medicated by acting on the αVβ3/5-FAK-STAT3 signaling axis. Combined with the basis of previous studies, we speculated that exogenous MFG-E8 may alleviate ER stress through activating the FAK-STAT3 signal axis, leading to subsequent improvement of mitochondrial function, reduction of oxygen free radical production, and ultimately protection of pancreatic exocrine acinar cells from damage.

Excessive ER stress activates inflammatory responses (Fritz et al., 2011; Yong et al., 2021). During ER stress, IRE1α recruits TRAF2 (TNF receptor-associated factor 2) to the ER membrane to initiate inflammatory responses via the NF-κB pathway (Urano et al., 2000). The activation of the inflammatory response in pancreatic tissue is accompanied by the release of a large number of inflammatory mediators into the blood, resulting in systemic inflammatory response syndrome (SIRS) (Ren et al., 2019b). In the current study, we also examined the effect of exogenous MFG-E8 on the inflammatory response, and found that the level of circulating inflammatory mediators decreased with the treatment of MFG-E8. These results suggest that intraperitoneal injection of exogenous MFG-E8, which was previously found to reduce overall mortality in a mouse model of acute pancreatitis (Ren et al., 2021a), may inhibit local and systemic inflammatory responses via suppressing ER stress. The inhibition of ER stress also reduces oxygen free radical production by improving the function of mitochondria.

Abnormal ER function is a powerful inducer of the transcription factor C/EBP homologous protein (CHOP). All three signaling pathways induced by unfolded protein response eventually induce the expression of CHOP, resulting in the cleavage of procaspase-12 under ER stress conditions and then activate caspases-9 and -3, accelerating cells into the programmed death phase (apoptosis) (Li et al., 2014; Datta et al., 2018). Several studies suggest that inducing apoptosis of damaged acinar cells effectively prevents the release of pancreatic enzymes caused by cell necrosis (Ren et al., 2019c; Zhang et al., 2021). However, excessive cell apoptosis will lead to irreversible loss of pancreatic parenchyma, resulting in more serious consequences (Ren et al., 2021a). Exogenous MFG-E8 not only reduced the expression level of CHOP, but also reduced the amount of apoptosis and necrosis of AR42J cells. These results indicate that MFG-E8 alleviates the cellular and extracellular damage during acute pancreatitis from multiple dimensions. The conclusion of this study is also a strong supplement and improvement of our previous study on exogenous MFG-E8 and mitochondrial injury in acute pancreatitis (Ren et al., 2021a).

There are some limitations of the study. First of all, due to the lack of clinical samples, we were unable to verify our findings in patients with acute pancreatitis. The clinical significance of this study warrants further investigation. In this paper, we reveal that exogenous MFG-E8 reduces the inflammatory response and apoptosis in pancreatic tissue by inhibiting ER stress (Hanayama et al., 2002; Aziz et al., 2011). However, whether the anti-inflammatory effect of MFG-E8 and the promotion of macrophage phagocytosis of apoptotic cells are related to the regulation of ER function remains unknown. Several studies have shown that MFG-E8 can directly regulate the NF-κB pathway (Zhao et al., 2019; Gong et al., 2020; Lu et al., 2021). In this study, we also found that MFG-E8 downregulated the NF-κB pathway. However, whether the NF-κB pathway plays a major role in MFG-E8-mediated inhibition of ER stress in pancreatic exocrine acinar cells remains to be determined. Therefore, the specific mechanisms of various biological effects of MFG-E8 need to be further explored. On the other hand, our recent study found that MFG-E8 limits the activation of pancreatic stellate cells by inhibiting chaperone-mediated autophagy by controlling unfolded protein response (Ren et al., 2021b). Whether MFG-E8 also improves the autophagy function of pancreatic acinar cells by regulating the ER signaling pathway, and then participates in the repair of acute pancreatitis, remains to be further explored.

## Conclusion

MFG-E8 maintains cellular homeostasis by alleviating ER stress in pancreatic exocrine acinar cells. The beneficial effects of MFG-E8 appears to be mediated through activating the αVβ3/5 integrin-FAK-STAT3 signaling pathway. These findings may provide a new perspective to reveal the role of MFG-E8 in acute pancreatitis.

## Materials and Methods

### Experimental Animals and *In Vivo* Models

Male adult C57BL/6J mice purchased from Experimental Animal Center of Xi’an Jiaotong University (Xi’an, China) and MFGE8 knockout (*mfge8*-KO) mice purchased from Shanghai Model Organisms Center (Shanghai, China) were used in this study. The *mfge8*-KO mice were generated as described previously (Ren et al., 2021a). The mice were fed on a standard laboratory chow diet and housed in a temperature-controlled room on a 12-h light/dark cycle. The study protocol was approved by the Institutional Animal Care and Use Committee of the Ethics Committee of Xi’an Jiaotong University Health Science Center. Mice (8–10 weeks, 20–22 g) were randomly divided into groups with six mice in each group and given intervention at the same time.

Arginine-AP was induced by 2 hourly intraperitoneal injections of 4.0 g/kg L-arginine (Sigma Aldrich, Shanghai, China). Two hours after the last injection of L-arginine, normal saline (vehicle) or 20 μg/kg MFG-E8 (RD System, Inc. Minnesota, United States) were administered intraperitoneally. The doses of MFG-E8 used in this study were chosen on the basis of our previous publications in acute pancreatitis (Ren et al., 2021a). To determine the role of αvβ3/5 integrin in MFGE8’s effect in ER stress, cilengitide (20 mg/kg, SELLECK, Texas, United States), a specific αvβ3/5 integrin inhibitor (Stupp et al., 2014; Li et al., 2021), were administered through intraperitoneal injection at 1 h after the last injection of L-arginine. At 72 h after the first injection of L-arginine, mice were anesthetized with isoflurane and serum and pancreatic tissue samples were collected.

Cerulein + LPS-AP was induced by 7 hourly injections of cerulein (50 μg/kg). Lipopolysaccharide (LPS, 10 mg/kg, L8880, Solarbio, Beijing, China) was added to the last cerulein injection (Liu et al., 2017). 20 μg/kg-MFG-E8 was intraperitoneally injected into mice 0.5 h after the second injection of cerulein. 4 h after the final injection of cerulein (7th injection), the mice were sacrificed under isoflurane inhalation anesthesia and pancreas tissue was collected.

### Cell Culture and *In Vitro* Model

The pancreatic AR42J cells were purchased from Sciencell (zq0145) and cultured in Ham’s F-12K medium (PM150910C, Procell) with 20% fetal bovine serum (164210-100, Procell) in a humidified incubator at 37°C with 5% CO_2_ (Liu et al., 2017). AR42J cells (5 × 10^5^/well) were plated into six-well culture plates and incubate for 24 h. The AR42J cells are the most commonly used cell line for *in vitro* studies of acute pancreatitis (Sandoval et al., 2010; Szmola and Sahin-Toth, 2010; Lugea et al., 2017).

#### *In Vitro* Model-1

The AR42J cells were treated with 2.5 or 5 nM thapsigargin (S7895, SELLECK, Texas, United States) with or without 20 or 100 ng/ml MFG-E8 (2805-MF, RD System, Inc. Minnesota, United States) for 24 h. Protein homogenate was extracted for subsequent detection.

#### *In Vitro* Model-2

The AR42J cells were treated with 0.5 or 1 μM tunicamycin (B7417, APExBIO, Houston, United States) with or without 20 or 100 ng/ml MFG-E8 (2805-MF, RD System, Inc. Minnesota, United States) for 24 h. Protein homogenate was extracted for subsequent detection.

#### *In Vitro* Model-3

The AR42J cells were treated with 3 or 6 μg/ml brefeldin A (S7046, SELLECK, Texas, United States) with or without 20 or 100 ng/ml MFG-E8 (2805-MF, RD System, Inc. Minnesota, United States) for 24 h. Protein homogenate was extracted for subsequent detection.

#### *In Vitro* Model-4

The AR42J cells were treated with 100 nmol/L cerulein (C6660, Solarbio, United States) and 10 ng/ml Lipopolysaccharide (LPS) (L-8880, Solarbio, United States) with or without 20 or 100 ng/ml MFG-E8 (2805-MF, RD System, Inc. Minnesota, United States) for 24 h. In additional groups of AR42J cells, Cilengitide (5 ng/ml, SELLECK, Texas, United States), a specific αvβ3/5 integrin inhibitor or PF00562271 (S2672, SELLECK, Texas, United States), a specific FAK inhibitor or APTSTAT3-9R (S8197, SELLECK, Texas, United States), a specific STAT3 antagonist, were administered together with 100 ng/ml MFG-E8.

### Statistical Analysis

All measurement data are expressed as the mean ± standard error (SEM). The *t*-test or one-way ANOVA with the Student-Newman-Keuls test was used to analyze the differences between groups. All analyses were conducted with data statistics software GraphPad Prism version 8.0 (GraphPad Software, Inc., San Diego, CA, United States). *p* < 0.05 represented a significant difference.

Methods for Flow Cytometry Analysis, Enzyme-linked immunosorbent assay (ELISA), GRP78 Staining, Immunohistochemical Staining, Transmission Electron Microscopy and Western Blot Analysis are provided in the Supplementary Materials.

## Data Availability

The raw data supporting the conclusion of this article will be made available by the authors, without undue reservation.

## Abbreviations

AP, Acute Pancreatitis; AR42J, cells of the rat exocrine pancreas; ATF6, Activating Transcription Factor 6; CHOP, C/EBP homologous protein; eIF2α, eukaryotic initiation factor 2α; ELISA, Enzyme-linked immunosorbent assay; ER stress, Endoplasmic Reticulum stress; GRP78, glucose-regulated protein 78; HMGB-1, High Mobility Group Box 1; IκBα, inhibitor of NF-κB-α; IL-6, Interleukin-6; IRE1α, inositol-requiring enzyme 1 α; KO, knockout; LPS, Lipopolysaccharide; MFG-E8, milk fat globule EGF factor 8; MPO, myeloperoxidase; NF-κB p65, Nuclear Factor Kappa-B p65; PERK, PKR-like endoplasmic reticulum kinase; SIRS, Systemic Inflammatory Response Syndrome; SAP, severe acute pancreatitis; TNF-α, tumor necrosis factor-α; UPR, unfolded protein response; WT, wsild type.
