# Corrigendum: MFG-E8 maintains cellular homeostasis by suppressing endoplasmic reticulum stress in pancreatic exocrine acinar cells

**DOI:** 10.3389/fcell.2022.1121052

**Published:** 2023-01-06

**Authors:** Yifan Ren, Wuming Liu, Jia Zhang, Jianbin Bi, Meng Fan, Yi Lv, Zheng Wu, Yuanyuan Zhang, Rongqian Wu

**Affiliations:** ^1^ National Local Joint Engineering Research Center for Precision Surgery and Regenerative Medicine, Shaanxi Provincial Center for Regenerative Medicine and Surgical Engineering, First Affiliated Hospital of Xi’an Jiaotong University, Xi’an, China; ^2^ Department of General Surgery, The Second Affiliated Hospital of Xi’an Jiaotong University, Xi’an, China; ^3^ Department of Hepatobiliary Surgery, First Affiliated Hospital of Xi’an Jiaotong University, Xi’an, China; ^4^ Department of Oncology, The Second Affiliated Hospital of Xi’an Jiaotong University, Xi’an, China; ^5^ Department of Pediatrics, First Affiliated Hospital of Xi’an Jiaotong University, Xi’an, China

**Keywords:** pancreatic exocrine acinar cells, endoplasmic reticulum stress, acute pancreatitis, MFG-E8, αvβ3/5 integrins, FAK-STAT3 pathway

In the original article, there was a mistake in Figures 2, 3 as published. The TEM results in the Sham group in Figure 2A inadvertently used the Sham group of mfge8-KO mice. In Figure 3A, the image of MPO with Vehicle treatment was misused from pancreas in CD11b group. The corrected Figures 2, 3 and its caption appear below:

**FIGURE 2:**
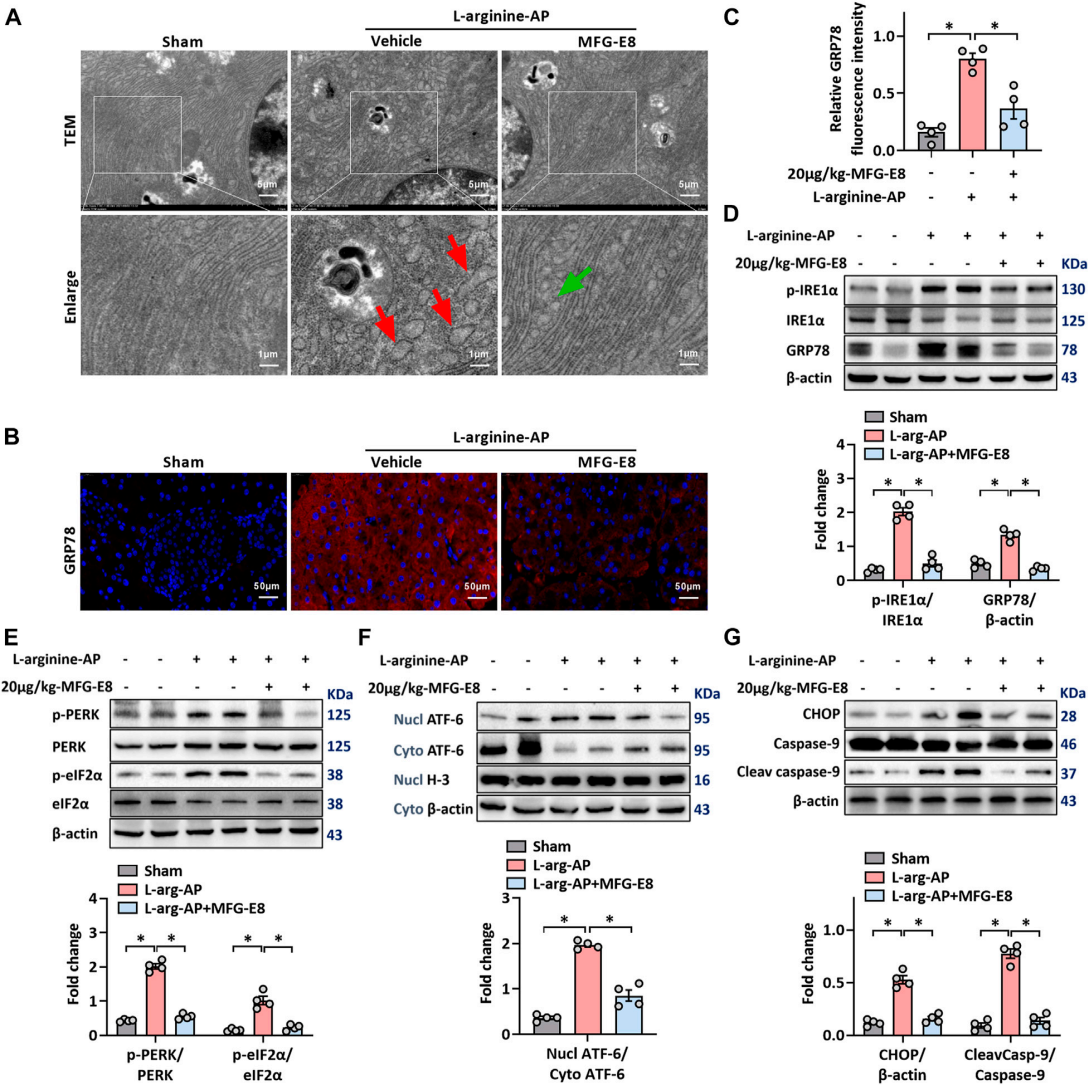
Exogenous MFG-E8 alleviates pancreatic ER stress *in vivo*. In mice, arginine-AP stress was induced by 2 h intraperitoneal injections of 4.0 g/kg L-arginine. At 2 h after the last injection of L-arginine, normal saline (vehicle) or 20 μg/kg MFG-E8 were administered through intraperitoneal injection. The animals were sacrificed at 69 h after MFG-E8 treatment (i.e., 72 h after the first injection of L-arginine). Blood and tissue samples were collected. **(A)** Ultrastructural alterations in the pancreas (Transmission electron microscopy); **(B,C)** Representative photos of GRP78 staining and quantitative of GRP78 staining; **(D)** Western blot analysis of the expression of GRP78, phospho-IRE1α and IRE1α in the pancreas; **(E)** Western blot analysis of the expression of phospho-PERK, PERK, phospho-eIF2α and eIF2α in the pancreas; **(F)** Western blot analysis of the expression of nucl-ATF-6, cyto-ATF-6, nucl-H3 and cyto-β-actin in the pancreas; **(G)** Western blot analysis of the expression of CHOP, caspase-9 and cleaved caspase-9 in the pancreas. *n* = 4–6/group, error bars indicate the SEM; ∗ *p* < .05 *versus* Sham group; #*p* < .05 *versus* Vehicle group. MFG-E8, milk fat globule EGF factor 8; AP, acute pancreatitis; GRP78, glucose-regulated protein 78; eIF2α, eukaryotic initiation factor 2α; ATF-6, Activating Transcription Factor 6; CHOP, C/EBP homologous protein; Nucl, nucleus; Cyto, cytoplasm; H-3, histone-3; PERK, PKR-like endoplasmic reticulum kinase.

**FIGURE 3:**
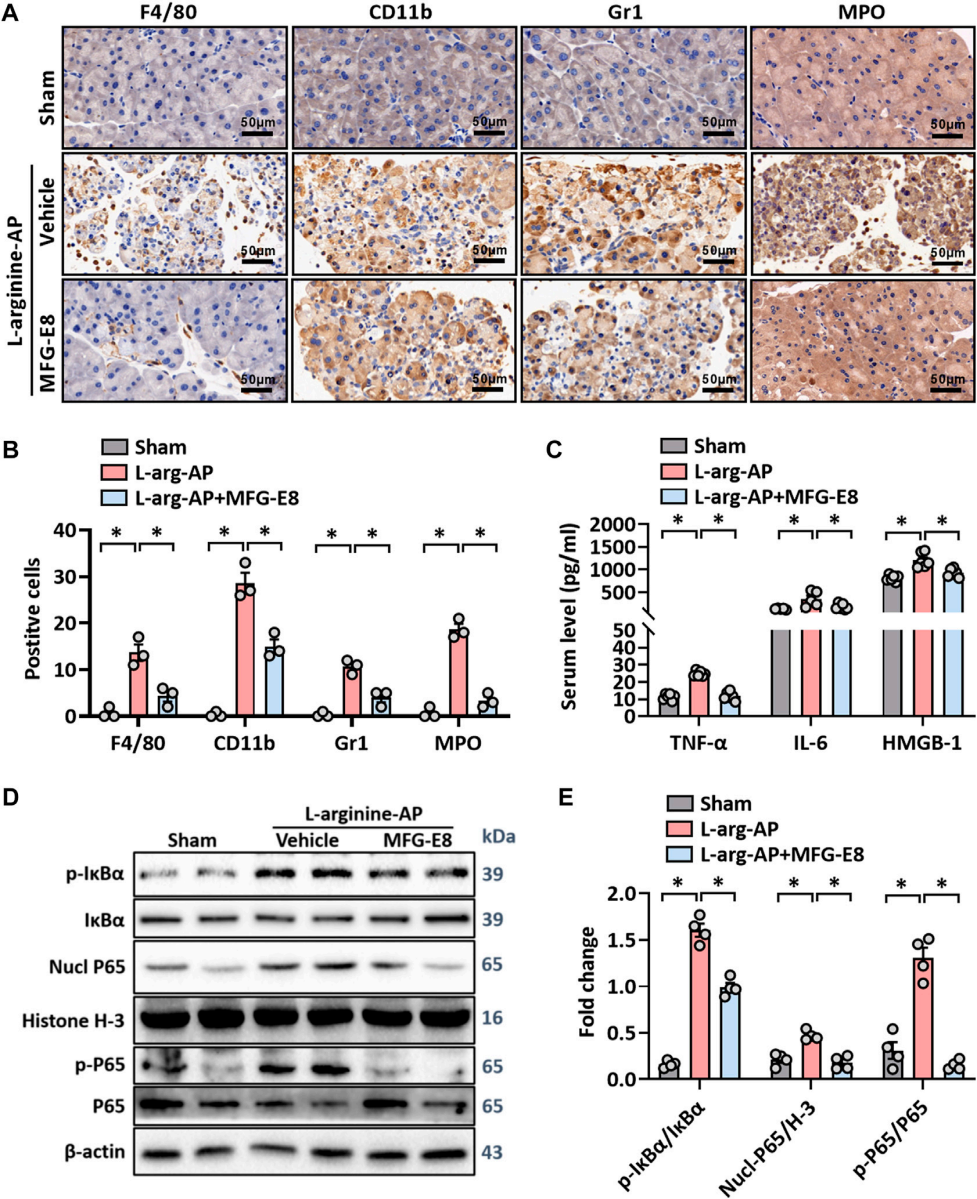
MFG-E8 alleviates the inflammatory response in experimental-AP through NF-κB signaling pathway. In mice, arginine-AP stress was induced by 2 h intraperitoneal injections of 4.0 g/kg L-arginine. At 2 h after the last injection of L-arginine, normal saline (vehicle) or 20 μg/kg MFG-E8 were administered through intraperitoneal injection. The animals were sacrificed at 69 h after MFG-E8 treatment (i.e., 72 h after the first injection of L-arginine). Blood and tissue samples were collected. **(A)** Representative photos of F4/80, CD11b, Gr1 and MPO staining; **(B)** Quantitative of F4/80, CD11b, Gr1 and MPO staining; **(C)** Serum TNF-α, IL-6 and HMGB-1 levels; **(D,E)** Western blot analysis of the expression of phospho-IκBα, IκBα, nucl-P65, cyto-P65, nucl-H3 and cyto-β-actin in the pancreas. *n* = 4–6/group, error bars indicate the SEM; ∗ *p* < .05 *versus* Sham group; #*p* < .05 *versus* Vehicle group. MFG-E8, milk fat globule EGF factor 8; AP, acute pancreatitis; MPO, myeloperoxidase; HMGB-1, High mobility group box 1; Nucl, nucleus; Cyto, cytoplasm; H-3, histone-3; IκBα, inhibitor of NF-κB-α; NF-κB p65, Nuclear Factor Kappa-B p65.

The authors apologize for this error and state that this does not change the scientific conclusions of the article in any way. The original article has been updated.

