# Supplementary material for: MFG-E8 Maintains Cellular Homeostasis by Suppressing Endoplasmic Reticulum Stress in Pancreatic Exocrine Acinar Cells
Source: Front Cell Dev Biol. 2022 Jan 14;9:803876. doi: 10.3389/fcell.2021.803876 (PMC8795834; doi:10.3389/fcell.2021.803876)
Supplement: Supplementary file 1 [file DataSheet1.docx]

Supplementary Materials for

**MFG-E8 Maintains Cellular Homeostasis by Suppressing Endoplasmic Reticulum Stress in**

**Pancreatic Exocrine Acinar Cells**

Yifan Ren, Wuming Liu, Jia Zhang, Jianbin Bi, Fan Meng, Yi Lv, Zheng Wu, Yuanyuan Zhang, Rongqian Wu.

*Corresponding author: [rwu001@mail.xjtu.edu.cn](mailto:rwu001@mail.xjtu.edu.cn)

**This file includes:**

Supplementary Method

Supplementary Table 1

Supplementary Figure 1

Supplementary Figure 2

Supplementary Figure 3

References

**Supplementary Method**

***Immunohistochemical staining:*** Pancreatic tissue from sacrificed animals was fixed in 4% formalin for paraffin embedding. As previously reported [1], paraffin sections were used for MPO, Gr1, CD11b and F4/80 staining, MPO antibody (ab45977, rabbit polyclonal, Abcam, USA) was used to mark the infiltration of neutrophils. Gr1 (LY6G) (ab25377, antibody, Abcam, USA), CD11b (ab216445, rabbit polyclonal, Abcam, USA) and F4/80 (ab240946, rabbit polyclonal, Abcam, USA) were used to demonstrate macrophage infiltration.

***Transmission Electron Microscopy (TEM):*** Seventy nm ultra-thin sections of pancreatic samples were stained with uranyl acetate and lead citrate. Pancreatic ultrastructure were evaluated using a transmission electron microscope (HT7700, Hitachi, Japan) by a single electron microscopist.

***Western Blot Analysis:*** Pancreas tissue was washed with ice-cold PBS and lysed by RIPA buffer at 4°C for 30 minutes. Total protein was collected by centrifugation at 14,000 rpm at 4°C for 20 minutes. Protein concentration was determined using the BCA method (P0009, Beyotime, Beijing, China). Protein was subjected to sodium dodecyl sulfate-polyacrylamide gel electrophoresis (SDS-PAGE) at 100 V for 2 hours and then transferred onto polyvinylidene fluoride (PVDF) membranes. The members were incubated with primary antibodies (Supplementary Table 1) at 4˚C overnight, and then secondary IgG-HRP antibody (goat anti-mouse IgG or goat anti-rabbit IgG, Pioneer Biotechnology, Shaanxi, China) was added onto membranes followed by incubation at 37˚C for 1 hour. Bands were developed using a digital gel image analysis system (Bio-Rad, California, USA), and the expression levels of proteins were calculated by ImageJ2x software as intensity relative to β-actin [2].

***Immunofluorescence Staining:*** For glucose-regulated protein 78 (GRP78) immunofluorescence staining, pancreatic tissue samples were fixed with 4% paraformaldehyde and then permeabilized with 0.5% Triton X-100. A primary rabbit anti-GRP78 antibody (Cell Signaling Technology, Beverly, MA, USA, 1:200 dilution) was incubated with samples overnight at 4°C. Alexa Fluor 594-conjugated Donkey Anti-Rabbit IgG(H+L) (SA00006-8, Proteintech, China, 1:200 dilution) was incubated for 1 h at room temperature.

***Enzyme-linked immunosorbent assay (ELISA):*** The mouse HMGB-1 ELISA kit (SEA399Mu, Cloud-Clone Corp USCN Life Science, Wuhan, China), IL-6 ELISA kit (SEA079Mu, Cloud-Clone Corp USCN Life Science, Wuhan, China) and tumor necrosis factor-α (TNF-α) ELISA kit (SEA133Mu, Cloud-Clone Corp USCN Life Science, Wuhan, China), were used for the detection of the levels of HMGB-1, IL-6 and TNF-α according to the manufacturer’s instructions.

**Supplementary Table 1: Antibodies**

| **Antibody** | **Item No** | **Company and location** |
| --- | --- | --- |
| CHOP (L63F7) Mouse mAb | 2895 | Cell Signaling Technology, Beverly, MA, USA |
| Anti-Caspase-9 antibody | ab184786 | Abcam, Cambridge, MA, USA |
| Cleaved-Caspase-9 Antibody | ABP50009 | Amyjet Scientific |
| FAK Antibody | 3285 | Cell Signaling Technology, Beverly, MA, USA |
| Phospho-FAK (Tyr576/577) Antibody | 3281 | Cell Signaling Technology, Beverly, MA, USA |
| Anti-β-actin | 60008 | proteintech, CN |
| Stat3 Rabbit mAb | 12640 | Cell Signaling Technology, Beverly, MA, USA |
| Phospho-Stat3 Antiboty | 9134 | Cell Signaling Technology, Beverly, MA, USA |
| Histone H3 (D1H2) | 4499 | Cell Signaling Technology, Beverly, MA, USA |
| ATF-6 (D4Z8V) Rabbit mAb | 65880 | Cell Signaling Technology, Beverly, MA, USA |
| GRP78 (C50B12) Rabbit mAb | 3177 | Cell Signaling Technology, Beverly, MA, USA |
| IRE1α (14C10) Rabbit mAb | 3294 | Cell Signaling Technology, Beverly, MA, USA |
| Anti-IRE1 (phospho S724) antibody | ab124945 | Abcam, Cambridge, MA, USA |
| eIF2α (D7D3) Rabbit mAb | 5324 | Cell Signaling Technology, Beverly, MA, USA |
| Phospho-eIF2α (Ser51) Rabbit mAb | 3398 | Cell Signaling Technology, Beverly, MA, USA |
| PERK (C33E10) Rabbit mAb | 3192 | Cell Signaling Technology, Beverly, MA, USA |
| Phospho-PERK (Thr982) Antibody | DF7576 | Affinity Biosciences, CN |
| IκBα (44D4) Rabbit mAb | 4812 | Cell Signaling Technology, Beverly, MA, USA |
| Phospho-IκBα (Ser32) Rabbit mAb | 5209 | Cell Signaling Technology, Beverly, MA, USA |
| Phospho-NF-κB p65 Rabbit mAb | 3033 | Cell Signaling Technology, Beverly, MA, USA |
| NF-κB p65 (D14E12) Rabbit mAb | 8242 | Cell Signaling Technology, Beverly, MA, USA |
| MFG-E8 antibody (F-5) | sc-271574 | Santa Cruz Biotechnology |
| Goat anti-Mouse IgG antibody | 31430 | PIONEER Biotechnology, CN |
| Goat anti-Rabbit IgG antibody | 31460 | PIONEER Biotechnology, CN |

**Supplementary figure 1.**

**
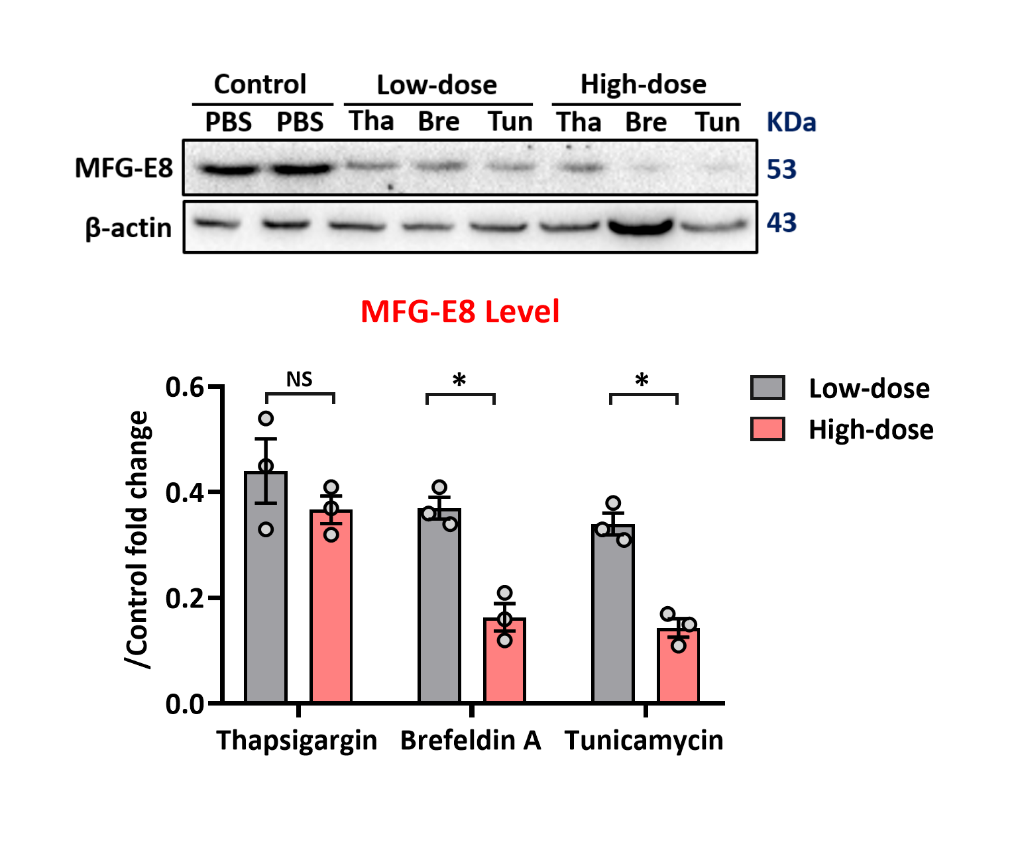
**

**The expression level of MFG-E8 decreased with the increase of the three ER stress activators.** *Model 1:* Pancreatic AR42J cells (5×10^5^/well) were treated with 2.5 or 5 nM thapsigargin for 24 hours. *Model 2:* Pancreatic AR42J cells (5×10^5^/well) were treated with 0.5 or 1 μM tunicamycin for 24 hours. *Model 3:* Pancreatic AR42J cells (5×10^5^/well) were treated with 3 or 6 μg/ml brefeldin A for 24 hours. Western blot analysis of the expression of MFG-E8 in the AR42J cells. n = 3/group, error bars indicate the SEM; ∗ P<0.05 versus Sham group. MFG-E8, milk fat globule EGF factor 8; GRP78, glucose-regulated protein 78; PBS, Phosphate Buffer Saline.

**Supplementary figure 2.**

**
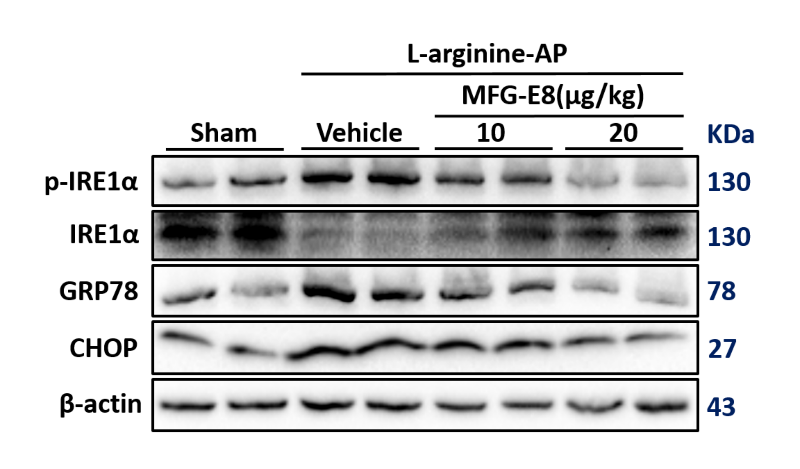
**

**Exogenous MFG-E8 alleviates pancreatic ER stress in vivo.** In mice, arginine-AP stress was induced by 2 hourly intraperitoneal injections of 4.0 g/kg L-arginine. At 2 hours after the last injection of L-arginine, normal saline (vehicle), 10 μg/kg or 20 μg/kg MFG-E8 were administered through intraperitoneal injection. The animals were sacrificed at 69 hours after MFG-E8 treatment (i.e., 72 hours after the first injection of L-arginine). Blood and tissue samples were collected. Western blot analysis of the expression of phospho-IRE1, IRE1α, GRP78 and CHOP in the pancreas. n = 2/group. MFG-E8, milk fat globule EGF factor 8; AP, acute pancreatitis; GRP78, glucose-regulated protein 78; CHOP, C/EBP homologous protein.

**Supplementary figure 3.**


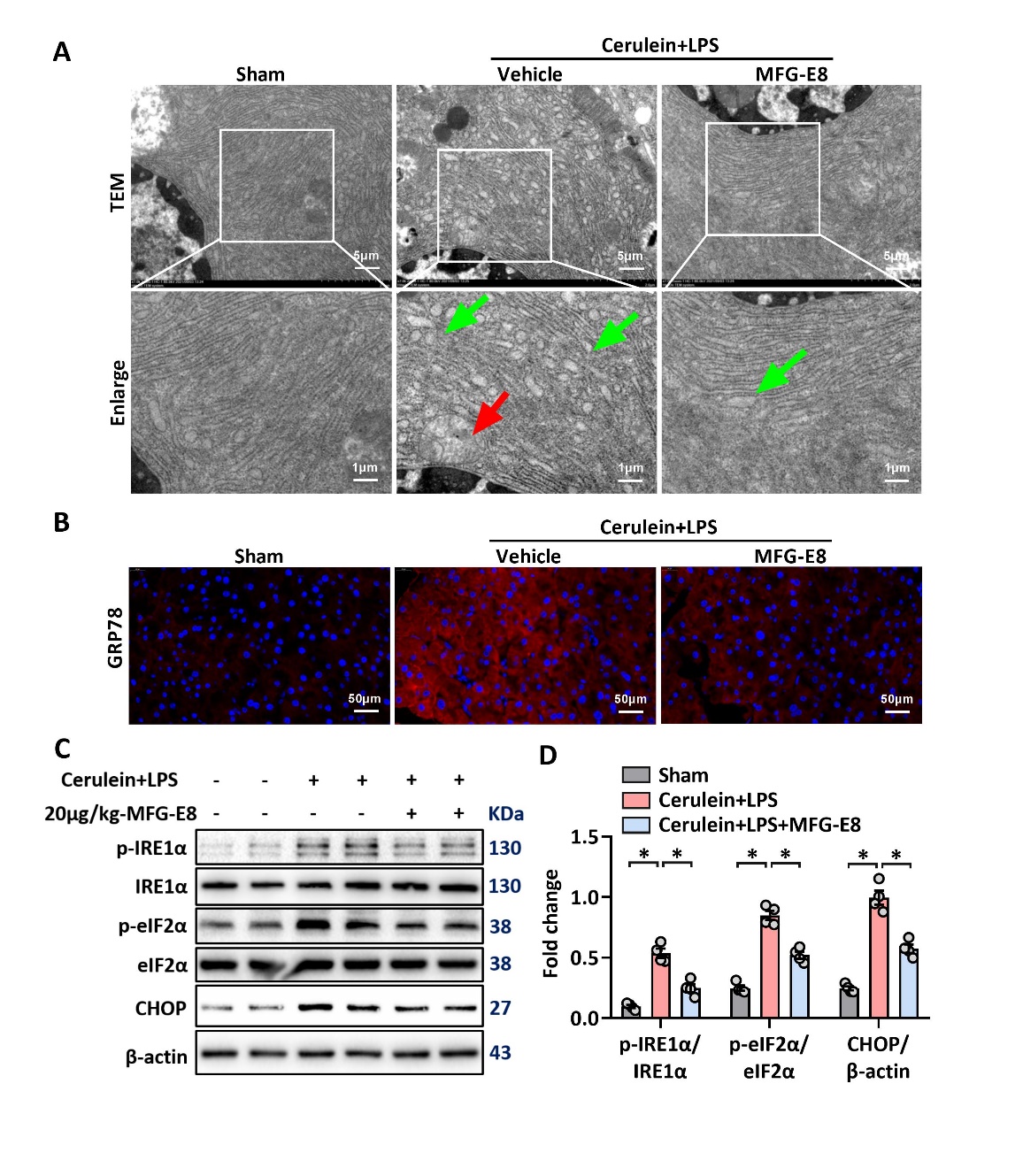


**MFG-E8 alleviated pancreatic ER stress in cerulein+LPS-treated mice.** Cerulein + LPS-AP was induced by 7 hourly injections of 50 μg/kg cerulein. LPS (10 mg/kg) was added to the last cerulein injection. At 30 min after the second injection of cerulein, 20 μg/kg MFG-E8 were administered through intraperitoneal injection. The animals were sacrificed at 4 h after the last injection of cerulein (ie, 11 h after the first injection of cerulein). Blood and tissue samples were collected. (**A**) Ultrastructural alterations in the pancreas (Transmission electron microscopy); (**B**) Representative photos of GRP78 staining; (**C&D**) Western blot analysis of the expression of phospho-IRE1, IRE1α, phospho-eIF2α, eIF2α and CHOP in the pancreas. n = 4-6/group, error bars indicate the SEM; ∗ P<0.05 versus Sham group; # P<0.05 versus Vehicle group. MFG-E8, milk fat globule EGF factor 8; LPS, lipopolysaccharide; GRP78, glucose-regulated protein 78; eIF2α, eukaryotic initiation factor 2α; CHOP, C/EBP homologous protein.

**REFERENCES**

1 Sendler M, Beyer G, Mahajan UM, Kauschke V, Maertin S, Schurmann C*, et al.* Complement Component 5 Mediates Development of Fibrosis, via Activation of Stellate Cells, in 2 Mouse Models of Chronic Pancreatitis. Gastroenterology 2015;**149**:765-76.e10.

2 Ren Y, Zhang J, Wang M, Bi J, Wang T, Qiu M*, et al.* Identification of irisin as a therapeutic agent that inhibits oxidative stress and fibrosis in a murine model of chronic pancreatitis. Biomed Pharmacother 2020;**126**:110101.
